# Pseudomyogenic hemangioendothelioma of bone with rare *WWTR1‐FOSB* fusion gene: Case report and literature review

**DOI:** 10.1002/ccr3.3808

**Published:** 2021-01-19

**Authors:** Khaled A. Murshed, Jorge Torres‐Mora, Ahmed Mounir ElSayed, Adham Ammar, Issam Al‐Bozom

**Affiliations:** ^1^ Department of Laboratory Medicine & Pathology Hamad Medical Corporation Doha Qatar; ^2^ Mayo Clinic Laboratories‐ Rochester Main Campus Rochester MN USA; ^3^ Department of Orthopedics surgery Hamad Medical Corporation Doha Qatar

**Keywords:** bone, *FOSB*, pseudomyogenic hemangioendothelioma, *WWTR1*

## Abstract

Pseudomyogenic hemangioendothelioma rarely arises in bone. *WWTR1‐FOSB* fusion gene is rarely reported in PMHE of bone. Currently, fusion genes can be used as diagnostic markers in PMHE; however, their prognostic and therapeutic significance is unclear.

## Graphical abstract

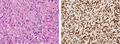

## 1 INTRODUCTION

Pseudomyogenic hemangioendothelioma (PMHE) is an uncommon vascular neoplasm of intermediate malignant potential that rarely arises in bone. *SERPINE1‐FOSB* fusion gene occurs frequently in PMHE of bone; however, *WWTR1‐FOSB* fusion gene is rarely reported. The prognostic and therapeutic significance of these gene rearrangements is unclear and needs to be investigated further.

Pseudomyogenic hemangioendothelioma (PMHE) is a rare endothelial neoplasm of intermediate malignant potential that usually arises in the soft tissues of the lower and upper extremities.^1^, ^2^, ^3^, ^4^ Its occurrence in bone is a rare event. To the best of our knowledge, only 27 cases of primary PMHE of bone have been reported so far ^4^, ^5^, ^6^, ^7^, ^8^, ^9^, ^10^, ^11^, ^12^, ^13^, ^14^, ^15^, ^16^, ^17^ (Table 1). Few of those reported cases were found to harbor the balanced translocation t(7;19)(q22;q13) producing fusion between *SERPINE1* and *FOSB* genes,^10^, ^12^ and only one case was found to carry *WWTR1‐FOSB* fusion gene.^17^ Herein, we present the second case of primary PMHE of bone with *WWTR1‐FOSB* fusion gene.

**TABLE 1:** Cases of Primary Pseudomyogenic Hemangioendothelioma of Bone

Case	Author (ref)	Year	Age	Sex	Site	Multifocal	Gene rearrangement
1	Hornick et al^4^	2011	35	Male	Upper extremity (finger)	No	Not performed
2	Sheng et al^5^	2012	10	Female	Left lower extremity	Yes	Not performed
3	McGinity et al^6^	2013	25	Male	Thoracic spine	No	Not performed
4	Righi et al^7^	2014	25	Male	Left upper extremity (distal radius)	No	Not performed
5	Righi et al^7^	2014	66	Female	Left lower extremity	Yes	Not performed
6	Shah et al^8^	2015	86	Male	Lower extremity	Yes	Not performed
7	Joseph et al^9^	2015	45	Male	Right pelvis (Ileum)	No	Not performed
8	Inyang et al^10^	2016	59	Male	Multiple	Yes	Not performed/failed
9	Inyang et al^10^	2016	19	Male	Right lower extremity	Yes	Not performed/failed
10	Inyang et al^10^	2016	47	Male	Left lower extremity	Yes	Not performed/failed
11	Inyang et al^10^	2016	14	Male	Left upper extremity	Yes	t(7;19)(q22;q13) with SERPINE1‐FOSB fusion
12	Inyang et al^10^	2016	74	Male	Spine and pelvis	Yes	Not performed/failed
13	Inyang et al^10^	2016	20	Male	Left lower extremity	Yes	Not performed/failed
14	Inyang et al^10^	2016	66	Male	Spine and pelvis	Yes	Not performed/failed
15	Inyang et al^10^	2016	12	Male	Left lower extremity	Yes	t(7;19)(q22;q13) with the SERPINE1‐FOSB fusion
16	Inyang et al^10^	2016	26	Male	Multiple	Yes	t(7;19)(q22;q13) with the SERPINE1‐FOSB fusion
17	Inyang et al^10^	2016	5	Female	Right lower extremity and pelvis	Yes	Not performed/failed
18	Ye et al^11^	2016	14	Female	Left lower extremity	Yes	Not performed
19	Ozeki et al^12^	2017	15	Male	Left lower extremity and spine	Yes	t(7;19)
21	Pradhan et al^13^	2018	9	Female	Lower extremity (proximal femur)	No	Failed
22	Pradhan et al^13^	2018	53	Male	Upper extremity (ulna)	Yes	Negative for t(7;19)
23	Pradhan et al^13^	2018	16	Male	Lower extremity (tibia)	No	Failed
24	Squillaci et al^14^	2018	46	Female	Right lower extremity (patella)	No	Not performed
25	Otani et al^15^	2019	20	Female	Left lower extremity	Yes	Not performed
26	Dianat et al^16^	2019	63	Male	Sacrum	No	Not performed
27	Panagopoulos et al^17^	2019	33	Female	Sacrum and spine	Yes	*WWTR1‐FOSB* fusion
28	Current case	2020	7	Female	Right lower extremity (distal femur)	No	*WWTR1‐FOSB* fusion

## 2 CASE PRESENTATION

**FIGURE 1:**
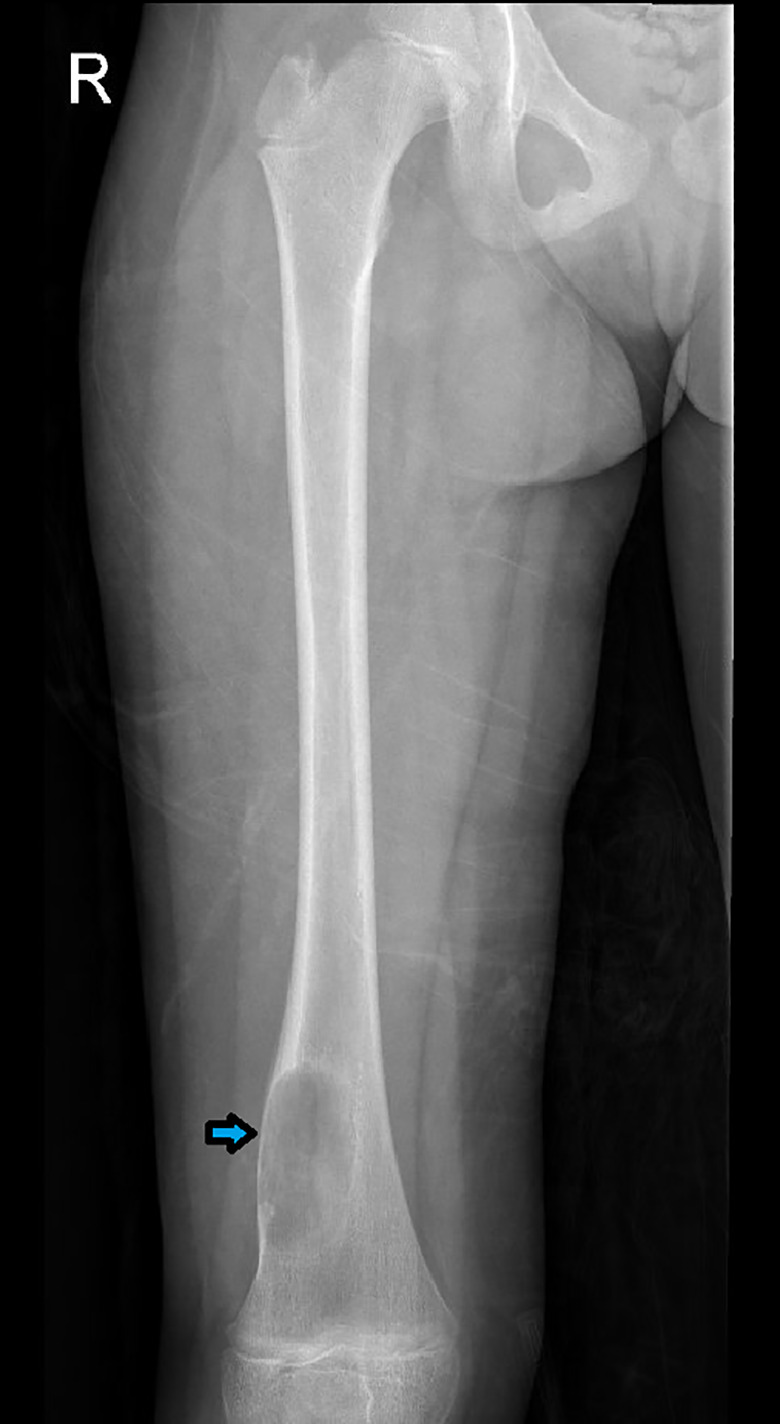
Plain X‐ray shows a well‐demarcated radiolucent lytic lesion in the metaphysis of the distal femur (blue arrow). There is no soft tissue involvement

**FIGURE 2:**
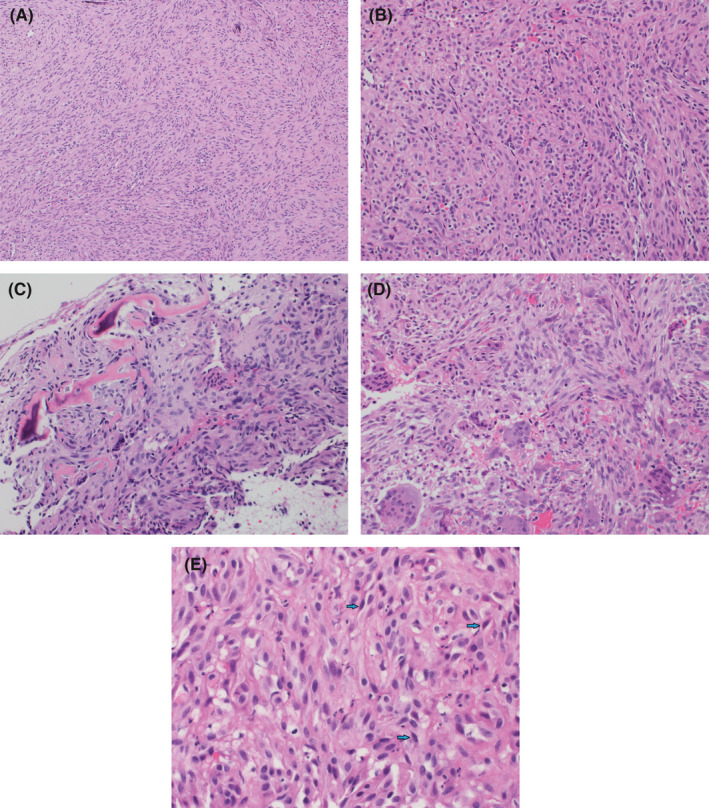
A, Photomicrograph shows sheets and fascicles of spindle tumor cells (hematoxylin and eosin stain, x100); B, mixture of plump spindle and epithelioid tumor cells (H&E stain, x200); C, the tumor with adjacent reactive woven bone (H&E stain, ×200); D, some areas show scattered osteoclast‐type multinucleated giant cells (H&E stain, ×200); and E, elongated tumor cells with dense eosinophilic cytoplasm (blue arrows) and prominent stromal neutrophils (H&E stain, ×400)

**FIGURE 3:**
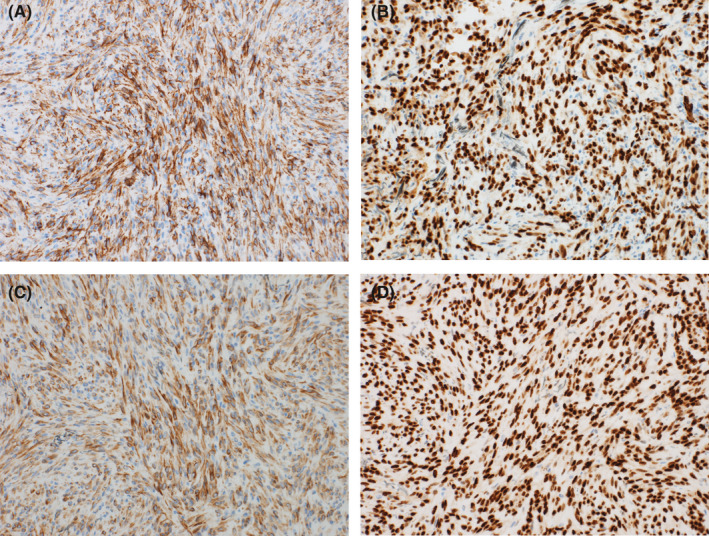
A, The tumor cells demonstrate immunoreactivity for CD31 antibody (immunohistochemistry, x200); B, diffuse nuclear staining for endothelial transcription factor ERG (IHC, ×200); C, diffuse staining for cytokeratin AE1/AE3 (IHC, x200); and D, diffuse and strong nuclear reactivity for FOSB antibody (IHC, ×200)

A previously healthy 7‐year‐old girl presented to the clinic with intermittent pain of the right thigh for two‐year duration. The pain was more severe at night. It was not associated with fever, weight loss, or other constitutional symptoms. Analgesics were given initially which relieved her symptoms temporarily; however, she started to feel pain at her right knee after an accident of falling. X‐ray was performed, which revealed a well‐demarcated radiolucent lytic lesion arising from the metaphysis of the right distal femur with cortical thinning. However, no periosteal reaction or soft tissue involvement was identified (Figure 1). A needle core biopsy was taken from the lesion. Histopathology shows a tumor composed of infiltrative sheets and fascicles of plump spindle and epithelioid cells (Figure 2A and 2B). In some areas, the tumor is admixed with reactive woven bone and osteoclast‐type multinucleated giant cells (Figure 2C and 2D). Some of the tumor cells are elongated and have dense eosinophilic cytoplasm with strap cell–like appearance (Figure 2E). Abundant numbers of neutrophils in the stroma are present. No stromal hyalinization or myxohyaline changes are identified. No vasoformative areas are seen. The tumor cells show mild nuclear atypia with few mitotic figures. However, there are no atypical mitoses or tumor cell necrosis. By immunoperoxidase stains, the tumor cells demonstrate reactivity for the vascular markers CD31 and ERG, and the epithelial marker cytokeratin AE1/AE3 (Figure 3A‐3C). They also show diffuse and strong nuclear reactivity for FOSB antibody (Figure 3D), but are negative for CAMTA1 and TFE3. A final diagnosis of pseudomyogenic hemangioendothelioma was rendered.

Next‐generation sequencing (NGS) was performed to identify possible targeted therapies for the patient's tumor. Genomic DNA was extracted from formalin‐fixed paraffin‐embedded (FFPE) tissue by macrodissection technique. A sarcoma‐targeted gene fusion panel was used to test for the presence of rearrangements in 138 targeted genes. The tumor was found to carry *WWTR1‐FOSB* fusion gene. Fusion/transcript variant junction locations and mutation nomenclature are based on RefSeq accession numbers: NM_015472 and NM_006732. The fusion/transcript variant junction location and the corresponding genomic coordinates within the *WWTR1* gene occur in exon 4 at genomic position Chr3:g.149260122 and within the *FOSB* gene occur in exon 2 at genomic position Chr19:g.45973887.

**FIGURE 4:**
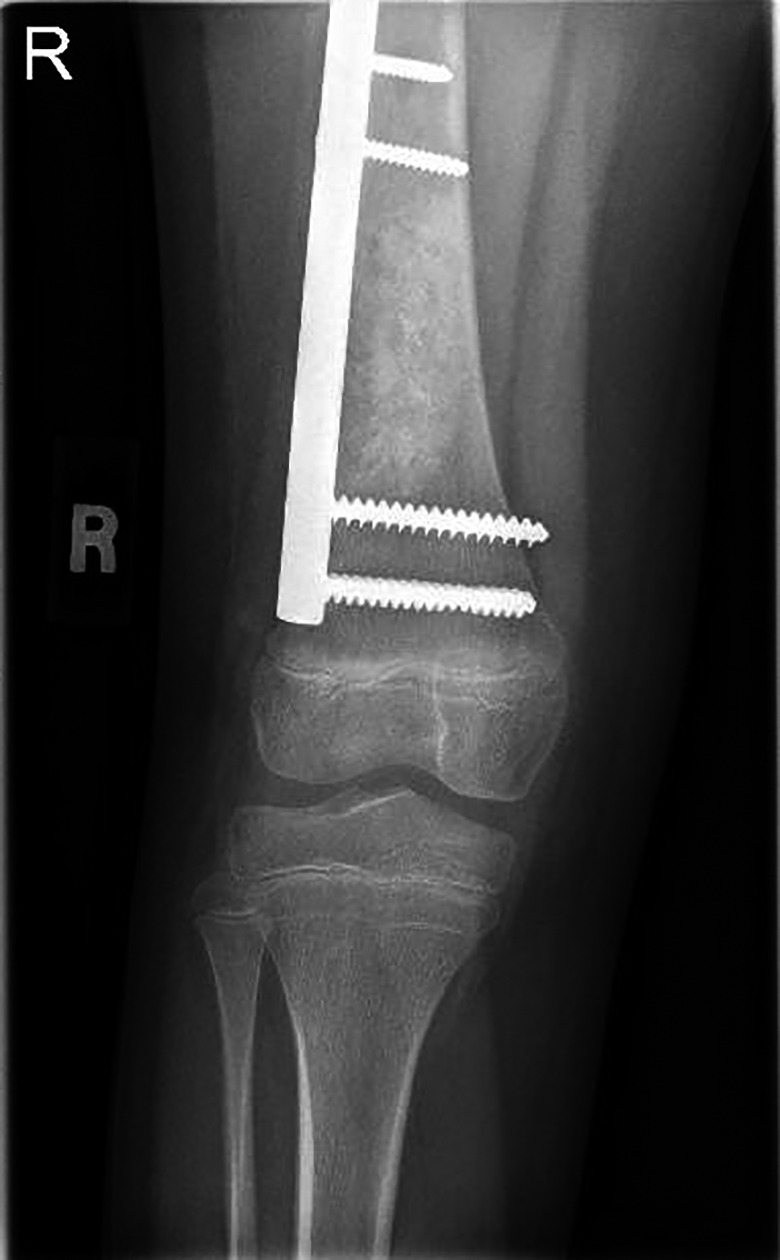
Postoperative knee X‐ray shows heterogeneous appearance of the distal right femoral shaft, and the site of bone grafting with intact plate and screw fixation

Whole‐body magnetic resonance imaging (MRI) and positron emission tomography (PET) scan were performed and revealed no evidence of distant metastasis. The patient underwent partial excision of the affected bone cortex with overlying muscle (vastus intermedius), along with extended curettage of the lesion. This was followed by filling of the defect using cancellous bone graft with internal fixation by plates and screws that do not cross the epiphyseal plate to avoid leg length discrepancy and growth retardation. Postoperative X‐ray showed satisfactory impaction of the bone graft and plate fixation (Figure 4). The patient was followed up for 6 months after operation, and there was no evidence of local recurrence during that period. She is still now on regular follow‐up.

## 3 DISCUSSION

PMHE is a rare vascular tumor with distinct clinical, pathologic, and molecular features. In 2003, the tumor was recognized as a distinctive entity under the name of “epithelioid sarcoma–like hemangioendothelioma” by Billing et al, due to combined features of morphological similarity with epithelioid sarcoma and immunoreactivity for vascular immunohistochemical markers.^1^ The term “pseudomyogenic hemangioendothelioma” was first proposed by Hornick and Fletcher in 2011, when they published a study of 50 cases of this tumor with extensive analysis of the clinicopathological and immunophenotypic features.^4^ The term was then adopted by the current World Health Organization (WHO) classification of tumors of soft tissue and bone in 2013.^2^

PMHE usually affects young adult males with a predilection for the lower limb. Multifocal presentation is not uncommon. However, in our case the tumor was unifocal. Morphologically, PMHE is an infiltrative neoplasm formed by sheets and loose fascicles of plump spindle and epithelioid cells having abundant bright eosinophilic cytoplasm. The tumor cells usually exhibit mild nuclear pleomorphism with vesicular nuclei and prominent nucleoli. Some cells have rhabdomyoblast‐like appearance with dense eosinophilic cytoplasm and peripherally located nuclei. Occasionally, prominent stromal neutrophils are present in the stroma.

PMHE is a locally aggressive tumor with high risk for local recurrence following surgical excision; however, it is a rarely metastasizing neoplasm.^1^, ^2^, ^3^, ^4^ It is essential to differentiate this tumor from other epithelioid soft tissue neoplasms including epithelioid hemangioma (EH), epithelioid hemangioendothelioma (EHE), epithelioid sarcoma (ES), and epithelioid angiosarcoma (EAS). Unlike PMHE, EH is characterized by well‐defined proliferation of vascular spaces lined by bland epithelioid endothelial cells with hobnailing into the lumen. The stroma usually contains varying numbers of eosinophils. Although the tumor cells in EH can show nuclear reactivity for FOSB antibody, cytokeratin AE1/AE3 is usually negative or shows patchy weak positivity. There is a considerable overlap between PMHE and the spindle cell variant of EH of bone.^18^, ^19^ Both tumors share similar clinical presentation, morphology, and immunophenotype, which make the distinction between them difficult and challenging. Both tumors commonly present as multifocal lesions, have prominent spindle cell component, and demonstrate immunoreactivity for cytokeratin and FOSB.^19^ Currently, the main distinguishing feature is the ability to demonstrate vasoformative areas lined by “tombstone”‐like cuboidal endothelial cells, a finding that is compatible with spindle cell EH. We were not able to demonstrate vasoformative areas in our case.

The distinction between PMHE and EHE can be very challenging. EHE is composed of tumor cells that have characteristic intracytoplasmic lumina containing red blood cells, embedded in myxohyaline stroma. EHE is characterized by recurrent t(1;3)(p36.3;q25) translocation, resulting in *WWTR1‐CAMTA1* fusion gene, and another subset of cases harbor *YAP1‐TFE3* fusion gene.^13^, ^20^, ^21^ Therefore, the tumor cells in EHE demonstrate diffuse nuclear expression for CAMTA1 and less commonly TFE3 by immunohistochemistry.^21^ Unlike PMHE, FOSB is negative in EHE. ES lacks the fascicular growth pattern. The tumor cells in ES are immunoreactive for CD34 and epithelial membrane antigen (EMA), but negative for FOSB. The most essential feature is loss of INI‐1 nuclear expression in ES, which is intact in PMHE. The tumor cells in EAS are arranged in freely anastomosing vascular channels lined by markedly atypical tumor cells with frequent mitotic figures including atypical forms. FOSB is negative in EAS.^22^

PMHE has distinct molecular prolife. The balanced translocation t(7;19)(q22;q13) producing fusion of *SERPINE1* and *FOSB* genes is well documented.^10^, ^12^, ^13^, ^23^ This translocation, which has not been detected in any other bone or soft tissue tumor, results in fusion of *SERPINE1* and *FOSB* genes, which leads to strong expression of FOSB. This gene product can be detected by immunohistochemistry. In 2018, Agaram et al and Zhu et al reported some cases of PMHE with recurrent *ACTB‐FOSB* gene fusions.^24^, ^25^ Recently, Panagopoulos et al reported a case of a 33‐year‐old woman with multifocal PMHE involving the sacrum and spine with novel *WWTR1‐FOSB* fusion gene, which was the first case of primary PMHE of bone carrying this fusion gene.^17^ In our case, the tumor was unifocal and harbors similar fusion gene detected by NGS, which revealed a fusion between *WWTR1* gene located on exon 4 of chromosome 3 with *FOSB* gene on exon 2 of chromosome 19.

It is essential to mention that *WWTR1‐FOSB* fusion is not specific for PMHE. In a study performed by Huang et al, *FOS* gene rearrangement was found to be present in a third of epithelioid hemangioma (EH) cases across different locations and histologic variants with more prevalence in cellular EH and intraosseous lesions.^20^, ^26^ The fusion genes detected in EH in that study include *ZFP36‐FOSB* and *WWTR1‐FOSB*.

In summary, we are presenting the second case of primary PMHE of bone with *WWTR1‐FOSB* fusion gene. Several fusion genes have been detected in PMHE. All these fusion genes lead eventually to upregulation of *FOSB* transcription factor, which makes it a useful immunohistochemical diagnostic marker for PMHE. The relationship between the various gene rearrangements occurring in PMHE and the clinicopathological features as well as the biological behavior of the tumor is still unclear, and further research should be pursued.

## CONFLICT OF INTEREST

The authors declare that they have no competing interests.

## AUTHOR CONTRIBUTIONS

KAM: conceived and designed the idea, performed literature review, wrote the manuscript, and overall organized the case report. JTM: performed further immunohistochemical stains and molecular studies on the case. AME: provided clinical information. AA: reviewed the case and the manuscript. IAB: reviewed the manuscript and supervised the project.

## ETHICS APPROVAL AND CONSENT TO PARTICIPATE

The Institutional Review Board of the Medical Research Council, Hamad Medical Corporation, Qatar, reviewed the protocol and approved it under the number (MRC‐04‐20‐250). Informed consent has been waived by the Institutional Review Board (IRB).

## Data Availability

The data that support the findings of this study are available from the corresponding author upon reasonable request.
